# Early vertebrate origin of CTCFL, a CTCF paralog, revealed by proximity-guided shark genome scaffolding

**DOI:** 10.1038/s41598-020-71602-w

**Published:** 2020-09-03

**Authors:** Mitsutaka Kadota, Kazuaki Yamaguchi, Yuichiro Hara, Shigehiro Kuraku

**Affiliations:** 1 Laboratory for Phyloinformatics, RIKEN Center for Biosystems Dynamics Research (BDR), Kobe, Japan; 2 Present Address: Research Center for Genome and Medical Sciences, Tokyo Metropolitan Institute of Medical Science, 2-1-6 Kamikitazawa, Setagaya-ku, Tokyo 156-8506 Japan

**Keywords:** Genome, Molecular evolution, Epigenetics, Zoology

## Abstract

The nuclear protein CCCTC-binding factor (CTCF) contributes as an insulator to chromatin organization in diverse animals. The gene encoding this protein has a paralog which was first identified to be expressed exclusively in the testis in mammals and designated as *CTCFL* (also called *BORIS*). *CTCFL* orthologs were reported only among amniotes, and thus *CTCFL* was once thought to have arisen in the amniote lineage. In this study, we identified elasmobranch *CTCFL* orthologs, and investigated its origin with the aid of a shark genome assembly improved by proximity-guided scaffolding. Our analysis employing evolutionary interpretation of syntenic gene location suggested an earlier timing of the gene duplication between *CTCF* and *CTCFL* than previously thought, that is, around the common ancestor of extant vertebrates. Also, our transcriptomic sequencing revealed a biased expression of the catshark *CTCFL* in the testis, suggesting the origin of the tissue-specific localization in mammals more than 400 million years ago. To understand the historical process of the functional consolidation of the long-standing chromatin regulator CTCF, its additional paralogs remaining in some of the descendant lineages for spatially restricted transcript distribution should be taken into consideration.

## Introduction

The CCCTC-binding factor (CTCF) contains the C2H2 Zn finger-type DNA binding domains and plays a pivotal role in chromatin organization as an insulator in diverse metazoans^1,2^. In vertebrates, the genome-wide binding landscape of the CTCF protein has been characterized for mammals^3,4^, sharks^5^, and the lamprey^6^, but the property of its paralog, CTCFL (also called BORIS, brother of the regulator of imprinted sites), has not been well characterized in a molecular phylogenetic context. CTCFL was first identified in human and mouse as a protein that functions in the testis and binds to the known target DNA of the CTCF protein in vitro^7^. Their differential functions have been intensively investigated in germ cells and cancer cells mainly from epigenetic viewpoints^8–11^. Comparison of amino acid sequences between CTCF and CTCFL exhibits a high similarity in the Zn finger DNA binding domain while the homology was low in other regions^7^, i.e., in the C-terminal region indispensable for the insulator function of CTCF^12^. In contrast to the ubiquitously expressed *CTCF*^7^, the expression of *CTCFL* is restricted to the male testis, more specifically in the spermatocyte and the spermatogonia^7,13^. Concordantly, while the mice lacking *CTCF* are embryonically lethal as early as E4.5^14^, mice lacking *CTCFL* are viable with phenotypes only in the testis, showing the marked reduction of its size caused in part by the increased rate of apoptosis during spermatogenesis^15^. The report of *CTCFL* orthologs was long confined to mammals and lizards^16^ but more recently the orthologs were identified in birds, turtle, snakes, and crocodiles^6,17^. The gene expression patterns of *CTCFL* has been documented for only amniote species, and it is hypothesized that the testis-specific expression of *CTCFL* was established in the ancestor of the therian mammals^16^. This previous study concluded that *CTCFL* was duplicated in the lineage leading to amniotes, which however was based on molecular phylogeny inference that does not seem to have been optimized for addressing this question—employing nucleotide sequences without multiple substitutions taken into account. Later, this hypothesis was questioned by a more rigidly controlled phylogenetic analysis using amino acid sequences of more diverse vertebrates^6^, which suggested an earlier origin of *CTCFL* than the split between chondrichthyan and osteichthyan lineages. This evolutionary scenario would be more reliably corroborated with accumulating information from recent genome sequencing of chondrichthyans^5^. A typical solution for dating gene duplication in an early age of vertebrate evolution involves genome expansion, referred to as two-round whole genome duplications (WGDs)^18,19^. This event gave rise to multiple arrays of chromosomal regions containing a similar set of genes, termed conserved synteny^20^. This strategy of phylogenetic characterization has not been applied to CTCF or CTCFL genes.

Exploration of gene repertoire and epigenome regulation has been facilitated by the recent release of large-scale molecular-level resources for multiple shark species^5^. This study included the landscape of CTCF protein binding in the cloudy catshark and the bamboo shark, as well as the whole genome assembly of the latter species^5^ whose completeness and continuity are comparable or superior to those of a member of Holocephali, *Callorhinchus milii*, that stood long as the only chondrichthyan species with the sequenced genome^21^. While the *CTCF* orthologs have been characterized even in jawless and cartilaginous fishes^5,6^, the available resources have not allowed the identification of *CTCFL* orthologs outside amniotes.

In this study, we improved the quality of the existing bamboo shark genome assembly with long-range scaffolding to reliably identify a *CTCFL* ortholog and characterize its phylogenetic property based on conserved synteny spanning the flanking genomic regions. With a further effort to identify *CTCF* and *CTCFL* orthologs in more diverse vertebrates, we inferred molecular phylogeny and provided a rigorous assessment of its output. Our study, supported by novel identification of elasmobranch *CTCFL* orthologs, consolidated an early origin of *CTCFL* through WGD which was only ambiguously suggested previously^6^. Our tissue-by-tissue transcriptome data also supported an early establishment of the testis-associated expression documented earlier solely for mammalian *CTCFL*.

## Results and discussion

### Proximity-guided genome scaffolding of the bamboo shark

**Figure 1:**
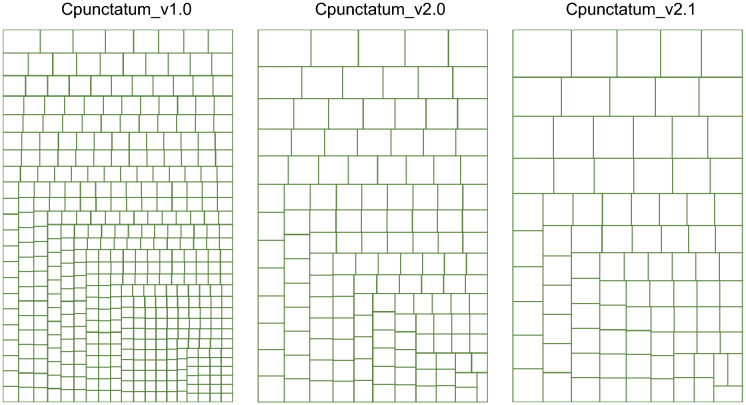
Treemap for comparing the continuity of the existing and improved brownbanded bamboo shark genome assemblies. Lengths of the genome scaffold sequences longer than the N50 scaffolding length of the individual assembly are shown with the sizes of the rectangles. The detailed properties of the individual genome assemblies are included in Table 1.

Previously, the whole genome shotgun reads and mate-pair reads of the brownbanded bamboo shark *Chiloscyllium punctatum* were assembled by the program Platanus^22^ to reconstruct its genome sequences^5^, which marked the N50 scaffold length of 1.96 Mbp (assembly version Cpunctatum_v1.0; NCBI Entry GCA_003427335.1). This assembly resulted from decontamination and a length cut-off at 500 bp for the Platanus output (see Methods of Ref.^5^). To improve the completeness and continuity of this assembly, we extracted high molecular weight genomic DNA extracted from the residual piece of the liver used for the production of the previously released assembly Cpunctatum_v1.0 (see “Methods” section). The genomic DNA was processed with in vitro chromatin reconstruction and proximity ligation to prepare two Chicago libraries (see “Methods” section), and they were sequenced to obtain 495 million read pairs in total. The obtained reads were used for long-range scaffolding by the program HiRise^23^. The scaffolding was performed in two separate runs with the minimum lengths for input sequences of 1,000 bp and 300 bp (versions Cpunctatum_v2.0 and Cpunctatum_v2.1, respectively), which both resulted in higher continuity than the input assembly that was previously released (version Cpunctatum_v1.0) as visualized in Fig. 1. Possibly because of the decreased cutoff length for the input sequences in scaffolding, the output with the cutoff of 300 bp (version Cpunctatum_v2.1) exhibited an increased continuity (N50 scaffold length, 9.19 Gbp; Table 1) and a larger maximum scaffold length (Fig. 1), which was adopted for the downstream sequence analysis in this study. This result emphasizes the importance of exploring different parameters in executing proximity-guided scaffolding, as shown previously for Hi-C scaffolding^24^.

**Table 1:** Improvement of the brownbanded bamboo shark genome assembly.

Metric	Cpunctatum_v1.0	Cpunctatum_v2.0	Cpunctatum_v2.1
N50 scaffold length (Kbp)	1,963	6,171	9,192
Max. length (Mbp)	17.15	38.70	56.09
Min. length (bp)	500	500	500
# scaffolds > 10 Mbp	14	72	82
# scaffolds > 1 Mbp	769	584	495
# scaffolds > 100 Kbp	2,797	1,372	1,253
# scaffolds > 10 Kbp	6,176	3,424	3,085
% gaps (‘N’)	9.83	10.05	10.39
# (%) of reference orthologs detected as ‘complete’	209 (89.70%)	210 (90.13%)	208 (89.27%)
# (%) of reference orthologs detected as ‘fragmented’	219 (93.99%)	221 (94.85%)	221 (94.85%)
# (%) of reference orthologs recognized as ‘missing’	14 (6.01%)	12 (5.15%)	12 (5.15%)

Sequences shorter than 500 bp are not taken into consideration. Gene space completeness was estimated by BUSCO v3 with the CVG, a set of 233 single-copy reference orthologs^36^.

### Identification of shark CTCF relatives

**Figure 2:**
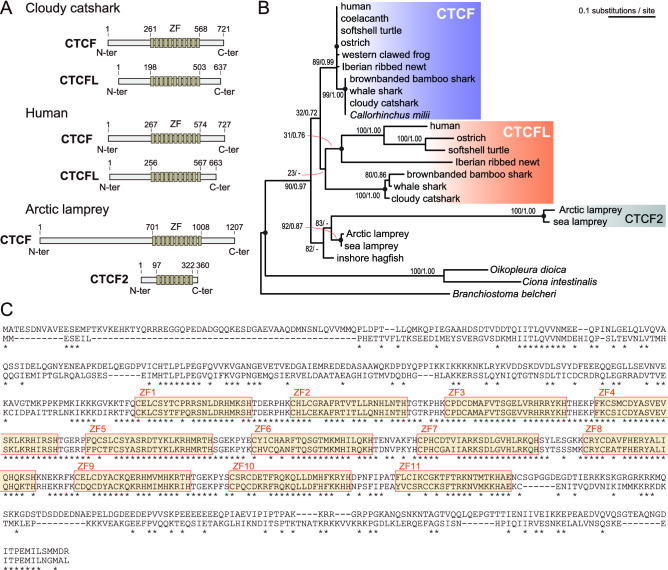
Structural and phylogenetic properties of the shark CTCF homologs. (**A**) Protein domain structures of the cloudy catshark CTCF and CTCFL in comparison with their homologs of human (CTCF and CTCFL) and Arctic lamprey (LjCTCF and LjCTCF2). The Zn finger domains (ZF) were identified by the webserver MOTIF Search (https://www.genome.jp/tools/motif/). (**B**) Molecular phylogenetic tree of the *CTCF* genes and their relatives. The tree was inferred with the maximum-likelihood method using 230 aligned amino acid sites. The support values at nodes indicate bootstrap values and posterior probabilities based on the maximum-likelihood method and Bayesian inference in order, respectively. See “Methods” section for details. (**C**) Pairwise amino acid sequence alignment of the cloudy catshark CTCF (top) and CTCFL (bottom). The alignment was generated by MAFFT^28^ ver. 7.471 by the iterative refinement method (L-INS-i). An asterisk indicates an identical amino acid residue. ZFs (1–11) identified by MOTIF Search are indicated with colored boxes. See Supplementary Fig. S2 for multiple alignment including more species.

Previously, we reported a putative full-length open reading frame (ORF) of the cloudy catshark (*Scyliorhinus torazame*) *CTCF* (NCBI GenBank: KY883979 including the ORF of Scyto0007366). An additional cloudy catshark *CTCF* homolog was identified by a BLASTP search in the deduced amino acid sequences of the cloudy catshark genes predicted on its whole genome assembly Storazame_v1.0 (GCA_003427355.1) using the amino acid sequence of the human *CTCFL* gene (NP_001255969.1). This search resulted in the highest bit score for the gene Scyto0009998 predicted on the genome scaffold scf_scyto00004224 whose sequences are different from those of the cloudy catshark CTCF (Supplementary Fig. S1). We also identified transcript contigs derived from our RNA-seq data^5^ that have overlapping nucleotide sequences to a part of Scyto0009998. One of the transcript contig sequences included a putative upstream region that partially matched the genome scaffold scf_scyto00086509, while the other two included a potential 3′ untranslated region (UTR). Using oligonucleotide primers designed in the potential 5′ and 3′ UTR of the putative second *CTCF* homolog, we amplified a fragment of cDNA reverse transcribed with the total RNA extracted from the adult testis. The 2,264 nt-long nucleotide sequence covering the whole putative ORF (637 amino acids, compared with its shorter predicted ORF of Scyto0009998 with 558 amino acids) was deposited as the entry KY883980 in NCBI GenBank. This gene is tentatively designated as the cloudy catshark *CTCFL* gene. We also identified potential orthologs of *CTCFL* in the whale shark and the brownbanded bamboo shark whose sequences are distinct from those of their *CTCF* orthologs (Rhity2000076 and Chipu0005442), respectively. The putative ORFs of these shark *CTCFL* genes, as well as their *CTCF* genes (Supplementary Data 1), were all revealed to possess eleven zing finger domains, as known for *CTCF* and *CTCFL* genes of osteichthyans including the human (Fig. 2A, C). The ORF lengths of the shark CTCF and CTCFL respectively resembled those of the mammalian counterparts rather than the lamprey homologs (Fig. 2A). The amino acid sequences of *CTCFL* orthologs (whose phylogenetic classification is confirmed below) exhibited a much lower similarity among them, compared with the *CTCF* counterparts, which is featured by the absence of the YDF motif in the amino acid sequences of *CTCFL* orthologs, with which CTCF interacts with cohesion and contributes to the formation of CTCF-anchored chromatin loops^25^ (Supplementary Fig. S2).

### Phylogenetic relationships among vertebrate CTCF relatives

We previously showed orthology of elasmobranch *CTCF* genes to osteichthyan *CTCF* genes^5^. To infer the phylogenetic relationships including the newly identified putative elasmobranch *CTCFL* genes, we reconstructed phylogenetic trees of the *CTCF* gene family with the maximum-likelihood (ML) method and the Bayesian approach using the amino acid sequences of the zinc finger domains (see “Methods” section). We have also included the newly identified sequences of the putative Iberian ribbed newt *CTCF* and *CTCFL* orthologs in this analysis. The ML tree displayed phylogenetic proximity of the putative elasmobranch *CTCFL* genes to the tetrapod *CTCFL* genes indicating their orthologous relationship (Fig. 2B). The putative elasmobranch *CTCFL* and tetrapod *CTCFL* genes exhibited long branches in comparison with their counterpart *CTCF* genes, showing that the *CTCFL* gene accepted much more amino acid substitutions than the *CTCF* did. We also observed a large heterogeneity of branch lengths among the different lineages of *CTCFL* genes. Importantly, the phylogenetic proximity between the putative elasmobranch *CTCFL* and tetrapod *CTCFL* genes were poorly supported in this ML tree (bootstrap value, 23; posterior probability, < 0.50).

To dissect the ambiguity in the phylogenetic relationship of the putative elasmobranch *CTCFL* genes in more detail, we performed an exhaustive likelihood computation for all possible tree topologies (see “Methods” section for details). In this analysis, internal relationships within several major operational taxonomic unit (OTU) (e.g., with osteichthyan *CTCF* genes) are constrained. The computed likelihoods are shown in Table 2, in which top ten tree topologies are listed in the descending order of the log-likelihoods, followed by the tree topologies exhibiting the largest log-likelihoods with the elasmobranch *CTCFL* or chondrichthyan *CTCF* (including the *Callorhinchus milii CTCF*) proximally clustering with either remaining OTU (Table 2). As a result, all tree topologies with the proximal cluster of the putative elasmobranch *CTCFL* with the chondrichthyan *CTCF* were statistically rejected by AU and Kishino–Hasegawa (KH) tests (*p* < 0.05; Table 2). In other words, chondrichthyan lineage-specific gene duplication between their putative *CTCFL* genes and the *CTCF* was not supported. On the other hand, proximal clustering of the putative elasmobranch *CTCFL* with the osteichthyan *CTCF* could not be rejected at the significance level of 0.05 in all the tests performed (e.g., Rank 416 in Table 2). Similarly, proximal clustering of the putative elasmobranch *CTCFL* with either of the lamprey *CTCF*, the lamprey *CTCF2*, or the hagfish *CTCF* remained unrejected (Rank 82, 101, and 163 in Table 2). Overall, regarding the phylogenetic position of the putative elasmobranch *CTCFL* genes, the molecular phylogenetic analysis did not provide unequivocal support, which prompted us to report to a different strategy, namely synteny analysis described below.

**Table 2:** Evaluation of tree topologies with the maximum-likelihood method.

Rank by ln*L*	Tree topology	*Δ*ln*L*	*p*AU (SE)^a^	*p*KH (SE)^b^	*p*SH (SE)^c^
1	(((Ost,Cho),(Tet-L,Ela-L)),((Lam,Lam-2),Hag),OG)	ML	0.954 (0.003)	0.691 (0.005)	1.000 (0.000)
2	((((Ost,Cho),Tet-L),Ela-L),((Lam,Lam-2),Hag),OG)	1.093172	0.837 (0.013)	0.309 (0.005)	0.996 (0.001)
3	(((Ost,Cho),(Tet-L,Ela-L)),(Lam,(Lam-2,Hag)),OG)	1.118902	0.782 (0.013)	0.237 (0.004)	0.994 (0.001)
4	(((Ost,Cho),(Tet-L,Ela-L)),((Lam,Hag),Lam-2),OG)	1.118911	0.685 (0.022)	0.237 (0.004)	0.994 (0.001)
5	((((Ost,Cho),Ela-L),Tet-L),((Lam,Lam-2),Hag),OG)	1.566155	0.491 (0.020)	0.246 (0.004)	0.989 (0.001)
6	((((Ost,Cho),Ela-L),Tet-L),((Lam,Lam-2),Hag),OG)	2.182305	0.593 (0.027)	0.234 (0.004)	0.988 (0.001)
7	((((Ost,Cho),Tet-L),Ela-L),((Lam,Hag),Lam-2),OG)	2.182311	0.593 (0.027)	0.234 (0.004)	0.988 (0.001)
8	(((Ost,Cho),((Lam,Lam-2),Hag)),(Tet-L,Ela-L),OG)	2.248093	0.507 (0.018)	0.186 (0.004)	0.983 (0.001)
9	((Ost,Cho),((Tet-L,Ela-L),((Lam,Lam-2),Hag)),OG)	2.248145	0.506 (0.018)	0.186 (0.004)	0.983 (0.001)
10	(((((Ost,Cho),(Tet-L,Ela-L)),Hag),Lam),Lam-2,OG)	2.325971	0.739 (0.016)	0.231 (0.004)	0.983 (0.001)
82	(((Ost,Cho),Tet-L),((Ela-L,Lam-2),(Lam,Hag)),OG)	8.286610	0.254 (0.056)	0.099 (0.003)	0.847 (0.004)
101	(((((Ost,Cho),Tet-L),Hag),(Ela-L,Lam)),Lam-2,OG)	9.115000	0.283 (0.029)	0.157 (0.004)	0.829 (0.004)
134	(((Ost,(Cho,Tet-L)),Ela-L),((Lam,Lam-2),Hag),OG)	10.332498	0.227 (0.043)	0.094 (0.003)	0.799 (0.004)
163	(((Ost,Cho),Tet-L),((Ela-L,Hag),(Lam,Lam-2)),OG)	11.212458	0.066 (0.027)	0.069 (0.003)	0.738 (0.004)
414	(((Ost,(Cho,Lam-2)),(Tet-L,Ela-L)),(Lam,Hag),OG)	15.082485	0.126 (0.016)	0.015 (0.001)	0.630 (0.005)
416	((((Ost,Ela-L),Cho),Tet-L),((Lam,Lam-2),Hag),OG)	15.177589	0.004 (0.009)	0.034 (0.002)	0.592 (0.005)
417	(((Ost,(Cho,Ela-L)),Tet-L),((Lam,Lam-2),Hag),OG)	15.177689	0.004 (0.009)	0.034 (0.002)	0.592 (0.005)
3,202	((((Ost,(Cho,Hag)),Tet-L),Ela-L),(Lam,Lam-2),OG)	37.521879	0.020 (0.006)	0.006 (0.001)	0.038 (0.002)
3,874	(((Ost,(Cho,Lam)),(Tet-L,Ela-L)),(Lam-2,Hag),OG)	48.528860	0.000 (0.000)	0.000 (0.000)	0.005 (0.001)

Cho, chondrichthyan CTCF; Ost, osteichthyan CTCF; Tet-L, tetrapod CTCFL; Ela-L, elasmobranch CTCFL; Lam, lamprey CTCF; Lam-2, lamprey CTCF2; Hag, hagfish CTCF; OG, outgroup; ln*L*, log-likelihood; *Δ*ln*L*, difference of log-likelihood deviated from the ML tree; SE, standard error of log-likelihood.

^a^*p *value of the AU test^32,37^.

^b^*p *value of the KH test^38^.

^c^*p *value of the Shimodaira-Hasegawa (SH) test^39,40^. The parentheses include standard errors. The underlined items in the tree topologies refer to the top-rank tree that supports their proximal clustering.

### Synteny analysis for the orthology between divergent CTCFL orthologs

**Figure 3:**
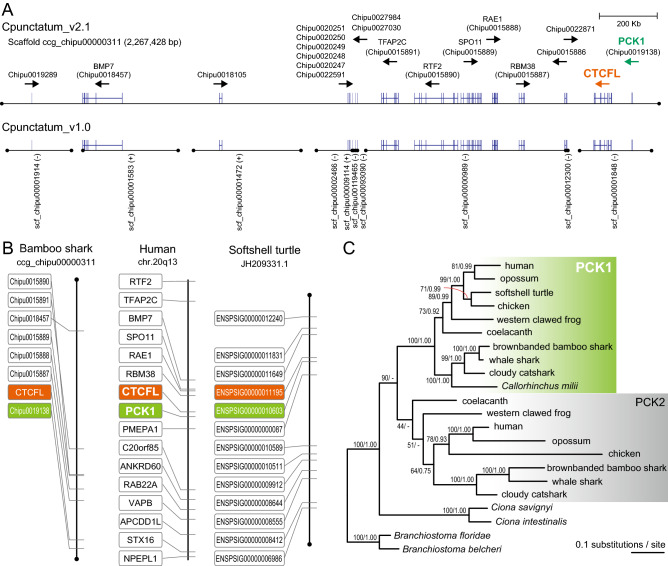
Synteny conservation in the genomic regions containing *CTCFL* orthologs. (**A**) Improved continuity of the bamboo shark genome assembly Cpunctatum_v2.1 by the Dovetail Chicago, in comparison with an earlier version Cpunctatum_v1.0. The ORF sequence of the *CTCFL* gene was derived from the scaffold ccg_chipu00000311 through manual curation. (**B**) Conserved synteny involving the *CTCFL* gene loci between human, softshell turtle, and bamboo shark. Only the orthologs that were confirmed by molecular phylogeny inference to be shared between the scaffold ccg_chipu00000311 of the bamboo shark genome assembly Cpunctatum_v2.1 and the human chromosome region 20q13 are shown, together with their orthologs of the scaffold JH209331.1 in the softshell turtle assembly PelSin1.0. Orthology is indicated with the same vertical level of the boxes. The *CTCFL* orthologs are indicated with orange boxes, and the *PCK1* orthologs (see **C**) with light green boxes. The black dots indicate scaffold ends. See Supplementary Fig. S3 for a genomic landscape for these species in which relative lengths between genes are taken into account. (**C**) Molecular phylogenetic tree of the *PCK1* gene and its relatives. The tree was inferred with the maximum-likelihood method using 616 aligned amino acid sites. The support values at nodes indicate bootstrap values and posterior probabilities based on the maximum-likelihood and Bayesian inference in order, respectively.

To investigate molecular phylogeny of putative *CTCFL* genes of elasmobranchs, we consulted possible synteny conserved across different vertebrate taxa. First, we employed the previously released cloudy catshark shark genome assembly Storazame_v1.0, only to find that it does not contain a scaffold sequence spanning more genes than the *CTCFL* ortholog (Supplementary Fig. S1). Therefore, we employed the previously released genome assembly of the brownbanded bamboo shark Cpunctatum_v1.0. In this genome assembly, the putative *CTCFL* gene was localized in the approximately 254 Kbp-long scaffold scf_chipu00001848, which however harbored one additional protein-coding gene (Fig. 3A). To overcome this situation, the abovementioned, newly built version of the genome assembly Cpunctatum_v2.1 was adopted for mapping this gene, which shows its localization in an approximately 2.3 Mbp-long scaffold ccg_chipu00000311 harboring seven additional predicted protein-coding genes (Fig. 3A, B).

We compared the composition of the genes flanking the putative bamboo shark *CTCFL* gene with two selected amniote species (the human and the softshell turtle) (Fig. 3B). Our molecular phylogenic analysis on the flanking genes supported the one-to-one orthology among these species, indicating that the gene array in those genomic regions is derived from the jawed vertebrate ancestor (Fig. 3C for the *PCK1* gene). Although our abovementioned phylogenetic analysis on *CTCF*/*CTCFL* did not provide unambiguous results, this observation of conserved synteny ascertains the orthology of the putative shark *CTCFL* genes with the previously identified amniote *CTCFL* genes (Fig. 3B).

### Synteny analysis for the paralogy between CTCF and CTCFL genes

**Figure 4:**
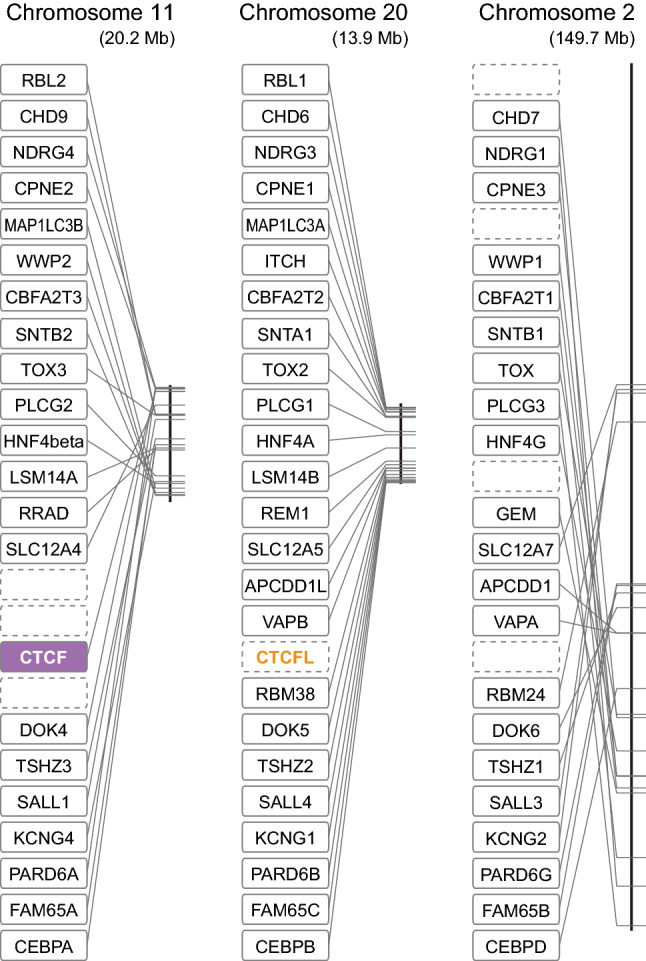
Large-scale chromosomal duplication between the *CTCF*-associated paralogons in the chicken genome. The diagonal lines show the positions of the genes in the boxes in the chicken genome assembly GRCg6a, while the vertical lines indicate the entire chromosomes, 11, 20, and 2 in order. The members of the same gene families that were confirmed by molecular phylogeny inference to be derived from two-round WGDs are aligned on the same vertical levels. The dashed boxes indicate the genes (including *CTCFL*) missing in this genome assembly probably because of its secondary loss during evolution.

Whereas the abovementioned synteny analysis scrutinized the orthology between *CTCFL* genes, the following analysis focuses on paralogy between *CTCF* and *CTCFL*. This analysis investigates whether these two genes arose in small-scale gene duplication or WGD whose timing is easier to pinpoint. In the human genome, the *CTCF* and *CTCFL* genes are located on chromosome 16 and 20, respectively, but the *CTCFL*-containing region is thought to have undergone frequent rearrangement of the gene order^26^. This prompted us to intensively analyze the homologous region in the chicken genome instead, although the chicken *CTCFL* ortholog is missing. In the chicken genome, the *CTCF* gene is localized in chromosome 11, while the genomic region from which the *CTCFL* ortholog was lost is localized on chromosome 20, still maintaining the neighboring genes. Between these chromosomes as well as the chicken chromosome 2, we observed quite a few gene families that have paralogs duplicated in early vertebrate evolution in common, such as *FAM65c*/*FAM65a*/*FAM65b* and *CHD9*/*CHD6*/*CHD7* (Fig. 4). This is consistent with the observation in the previous study based on genome-wide synteny analysis^26^. Although our analysis did not unveil the fourth chromosome or chromosome part that has maintained the equivalent gene array in the chicken genome, this observation, consistent with the previously documented pattern^18,19^, suggests that *CTCF* and *CTCFL* also split as a part of WGD rather than small-scale duplication. Altogether, our study shows that the *CTCF*-*CTCFL* duplication occurred around the emergence of vertebrates, as a part of the two-round WGDs.

### Asymmetric expression patterns between shark CTCF and CTCFL

**Figure 5:**
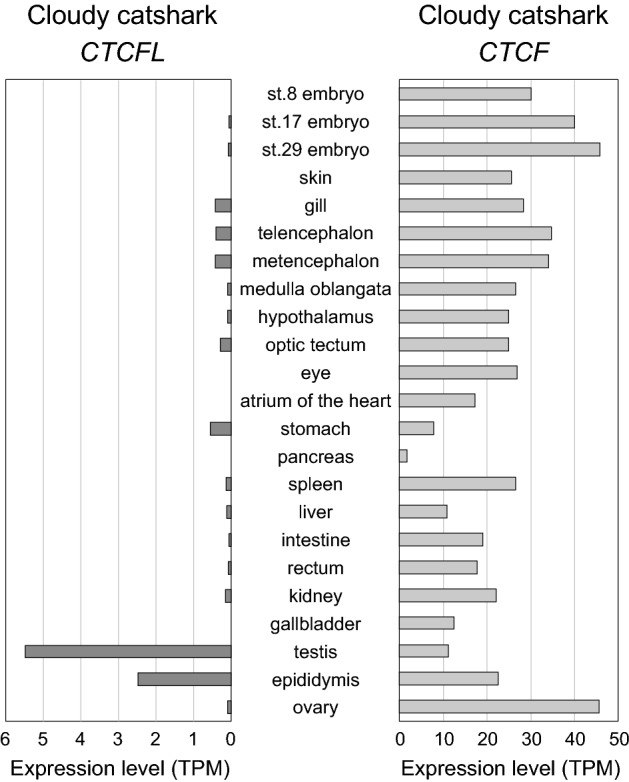
Expression profiles of *CTCF* and *CTCFL* in cloudy catshark tissues. Expression levels of cloudy catshark *CTCF* and *CTCFL* in adult tissues and embryos at different developmental stages were quantified in TPM (transcripts per kilobase million mapped reads) by the eXpress program using reads mapped to the coding nucleotide sequences of the cloudy catshark (see “Methods” section). Note that the scales are not equal between the genes. Cloudy catshark embryos were staged according to the existing literature^35^. The details of the RNA-seq data used for the analysis are included in Supplementary Table S2. The equivalent expression profiles of the brownbanded bamboo shark *CTCF* and *CTCFL* genes are included in Supplementary Fig. S4.

**Figure 6:**
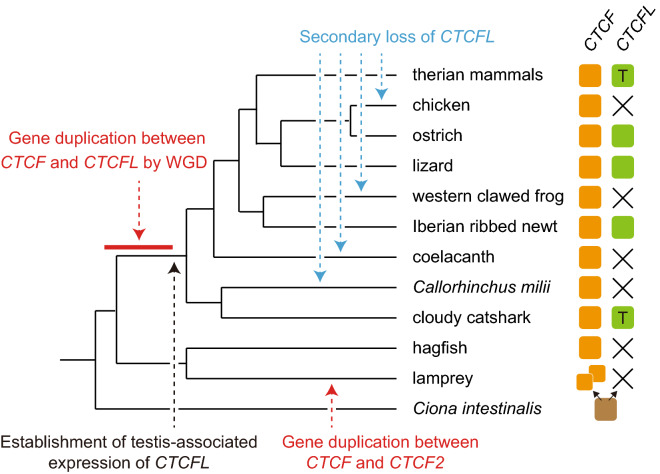
Evolutionary scenario of *CTCF* and *CTCFL* genes. Timings of gene duplication and loss are indicated with dashed arrows. Numbers of the colored boxes on the right show the number of genes in the genome, and the symbol ‘X’ indicates absence of the gene in the currently available genome assembly. The letter ‘T’ in the box of a *CTCFL* gene indicates its testis-specific gene expression.

To examine possible commonalities of expression patterns with mammals, we analyzed tissue distribution of shark *CTCF* and *CTCFL* expression. Using the RNA-seq data released previously^5^, we quantified their expression levels in embryos and adult tissues (Fig. 5). This analysis revealed an intensive expression of the *CTCFL* ortholog in the catshark testis, as described in mammals, while the *CTCF* ortholog is widely expressed. It should be noted that the catshark *CTCFL* is also expressed in the epididymis, whereas no equivalent expression has been documented for its mammalian ortholog^27^. It is suggested that the *CTCFL* ortholog was recruited for some role in the male reproductive organ before the split between the chondrichthyan and osteichthyan lineages. Later, at least the shark lineage, as well as the therian mammal lineage, have retained the testis-associated expression, while other lineages, including the chicken and anuran lineages, secondarily lost the *CTCFL* orthologs (Fig. 6).

## Conclusions

This study challenged the previous understanding of the timing of the duplication between *CTCF* and *CTCFL* (*BORIS*). By exploiting the emerging genome and transcriptome sequence information of formerly underrepresented taxa, we performed in-depth molecular phylogenetic analysis, reinforced by evolutionary interpretation of syntenic gene location. This investigation suggested that *CTCF* and *CTCFL* were duplicated earlier than previously thought, namely before the divergence between the osteichthyan and chondrichthyan lineages, possibly around the time of the occurrence of vertebrates (Fig. 6). Our analysis revealed testis-associated expression of the shark *CTCFL* orthologs, suggesting that the *CTCFL* was already intensively expressed in the testis at the osteichthyan-chondrichthyan divergence (Fig. 6). Altogether, the well-studied chromatin regulator CTCF has a complex evolutionary history, with its sister gene retained by some of the descendant gnathostome lineages with restricted expression domains.

## Methods

### Genomic DNA extraction and genome scaffolding with Dovetail Chicago

We used a residual piece of the liver dissected from the brownbanded bamboo shark *C. punctatum* individual used in our initial genome sequencing^5^, which was kept at − 80 °C for 15 months. Our study was conducted in accordance with the institutional guideline Regulations for the Animal Experiments and approved by the Institutional Animal Care and Use Committee (IACUC) of the Institute of Physical and Chemical Research (RIKEN) Kobe Branch (Approval ID: H16-11). The liver tissue of about 100 mg was homogenized with a dounce tissue grinder (Sigma Aldrich) on ice, followed by the addition of cold-ethanol solution to the final concentration of 50% for the fixation on ice for 1 h, and the resultant cell suspension was embedded in agarose gel. The agarose gel plugs were processed with the CHEF Mammalian Genomic DNA Plug Kit (BioRad, Cat. No. #1703591) to extract ultra-high molecular weight DNA. The processed agarose plugs were digested by the Agarase (Thermo Fisher Scientific, Cat. No. #EO0461), and the extracted DNA was purified by drop dialysis using the MF-Millipore Membrane Filter (Merck Millipore, Cat. No. # VCWP04700). Length distribution of the genomic DNA was analyzed by pulsed-field gel electrophoresis, which exhibited an average length of over 2 Mbp. Using the genomic DNA, two Chicago libraries were constructed, which were sequenced at Dovetail Genomics. Scaffolding with the program HiRise^23^ was performed twice using the Chicago sequencing data and the previously generated *C. punctatum* genome assembly which contains additional sequences shorter than 500 bp^5^. The cut-off lengths of input sequences in executing HiRise were set individually to 1,000 bp and 300 bp for Cpunctatum_v2.0 and Cpunctatum_v2.1, respectively.

### Molecular phylogenetic analysis

Protein sequences used for phylogenetic analysis were collected from the NCBI and Ensembl databases except those manually curated (Supplementary Data 1). The accession IDs of the sequences used for the phylogenetic analysis are included in Supplementary Table 1. The deduced amino acid sequences were aligned with the MAFFT^28^ v7.299b using the L-INS-i method. The aligned sequences were trimmed with trimAl^29^ v1.4.rev15 using the ‘-automated1’ option, followed by the removal of gapped sites using the ‘-nogaps’ option. The maximum-likelihood tree was inferred with RAxML^30^ v8.2.8 using the PROTCATWAG model, and for evaluating the confidence of the nodes, the rapid bootstrap resampling with 1,000 replicates was performed. Molecular phylogenetic tree employing the Bayesian framework was inferred with PhyloBayes^31^ v4.1 using the CAT-WAG-Γ model.

Evaluation of tree topologies (Table 2) was performed with CONSEL^32^ v1.20 and RAxML using the PROTGAMMAWAG model. For all possible tree topologies and statistical tests, the internal relationships of the sequences used in the phylogenetic analysis were constrained to the following eight groups at the locations of the black circles plotted at each node in Fig. 2B; osteichthyan CTCF, chondrichthyan CTCF, tetrapod CTCFL, putative elasmobranch CTCFL, lamprey CTCF, lamprey CTCF2, inshore hagfish CTCF, and the outgroup.

### Synteny analysis

Detection of conserved synteny was performed as described previously^20^. To identify the chromosome positions of the brownbanded bamboo shark genes, their coding nucleotide sequences (in the file ‘Cpunctatum_v1.0.cds.nuc.fna’ retrieved from https://doi.org/10.6084/m9.figshare.6124964.v1) predicted on the previous version of the genome assembly^5^ were mapped to the genome assembly Cpunctatum_v2.1 by the program BLAT v36. Phylogenetic properties of the genes located in the regions harboring the orthologs of *CTCF* or *CTCFL* as well as the regions homologous to them were analyzed by inferring molecular phylogenetic trees using the webserver aLeaves^33^ and the method described above. The selection of the candidate gene families for the phylogenetic analysis was assisted by the OHNOLOGS database (https://ohnologs.curie.fr)^34^.

### Gene expression quantification

We used the RNA-seq data of various cloudy catshark tissues produced in our previous study^5^. Gene expression levels were quantified as described previously^6^, except that mapping was performed by Bowtie2 v2.3.3.1 with the nucleotide sequence set of the predicted cloudy catshark genes (in the file ‘Storazame_v1.0.cds.nuc.fna’ retrieved from https://doi.org/10.6084/m9.figshare.6124664.v1), in which the sequences of Scyto0007366 and Scyto0009998 were replaced with those of KY883979 and KY883980 to assure the inclusion of the full coding sequences and the UTRs. The mapping result was processed with eXpress v1.5.1 to compute transcripts per million mapped reads (TPM).

## Supplementary information

Supplementary Information.

## Data Availability

Sequencing reads of the Dovetail Chicago libraries were deposited to the DNA Data Bank of Japan (DDBJ) under the accession number DRA009755. The brownbanded bamboo shark genome assemblies Cpunctatum_v2.0 and Cpunctatum_v2.1 are available at Figshare (https://figshare.com/projects/sharkgenome2-CTCFL/75273).
