# Supplementary material for: Early vertebrate origin of CTCFL, a CTCF paralog, revealed by proximity-guided shark genome scaffolding
Source: Sci Rep. 2020 Sep 3;10:14629. doi: 10.1038/s41598-020-71602-w (PMC7471279; doi:10.1038/s41598-020-71602-w)
Supplement: Supplementary file 1 — Supplementary Information. [file 41598_2020_71602_MOESM1_ESM.pdf]

Supplementary Information

Early vertebrate origin of CTCFL, a CTCF paralog, revealed by proximity-guided shark genome scaffolding

Mitsutaka Kadota[1†], Kazuaki Yamaguchi[1†], Yuichiro Hara[1**], and Shigehiro Kuraku[1*]

[1] Laboratory for Phyloinformatics, RIKEN Center for Biosystems Dynamics Research (BDR), Kobe, Japan

[†]These authors contributed equally

* Correspondence: shigehiro.kuraku@riken.jp

**Present address: Research Center for Genome & Medical Sciences, Tokyo Metropolitan Institute of Medical Science, 2-1-6 Kamikitazawa, Setagaya-ku, Tokyo 156-8506, Japan

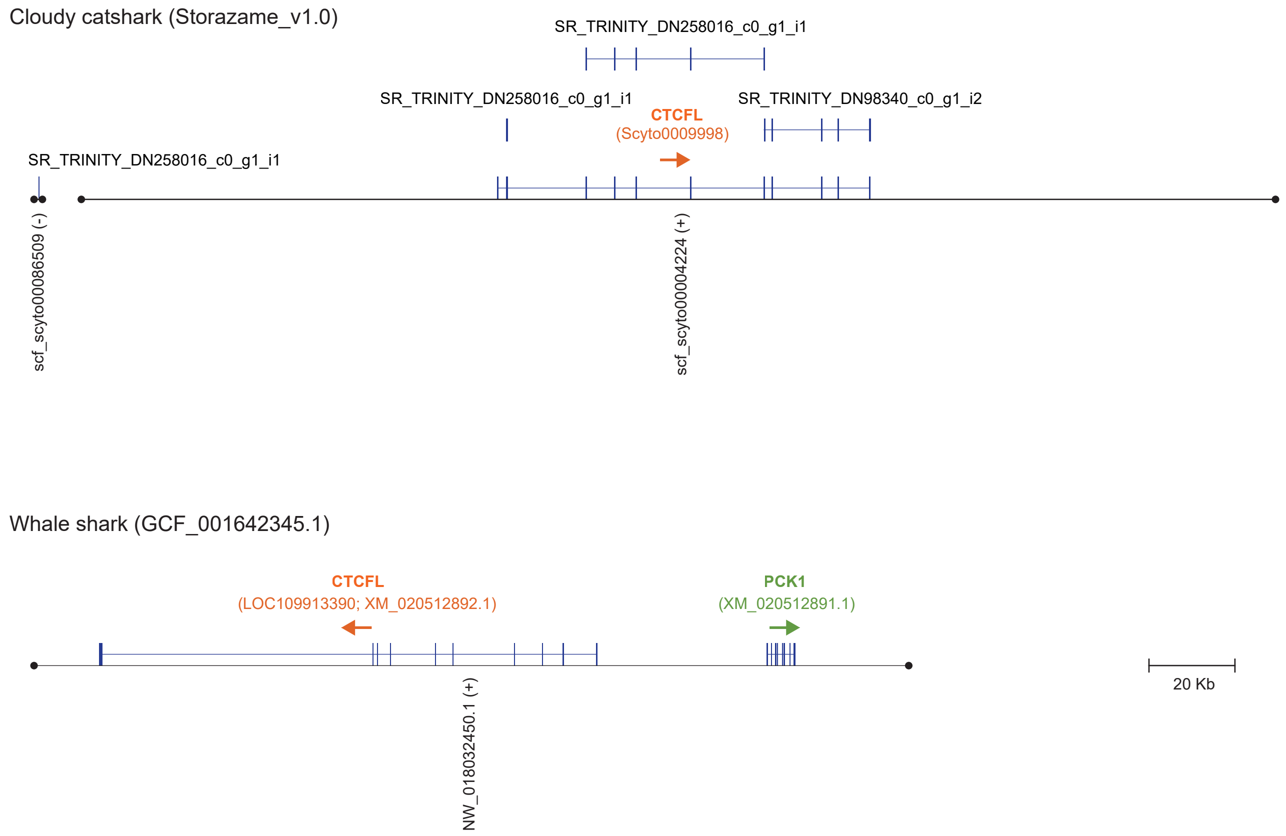

Supplementary Figure S1. *CTCFL*-containing genomic regions of cloudy catshark and whale shark. The *CTCFL*-containing scaffolds in the cloudy catshark genome assembly Storazame_v1.0 and the whale shark genome assembly GCF_001642345.1 are shown together with predicted genes and their transcript contigs obtained from the elasmobranch sequence archive Squalomix (https://transcriptome.riken.jp/squalomix/). The black dots indicate scaffold ends.

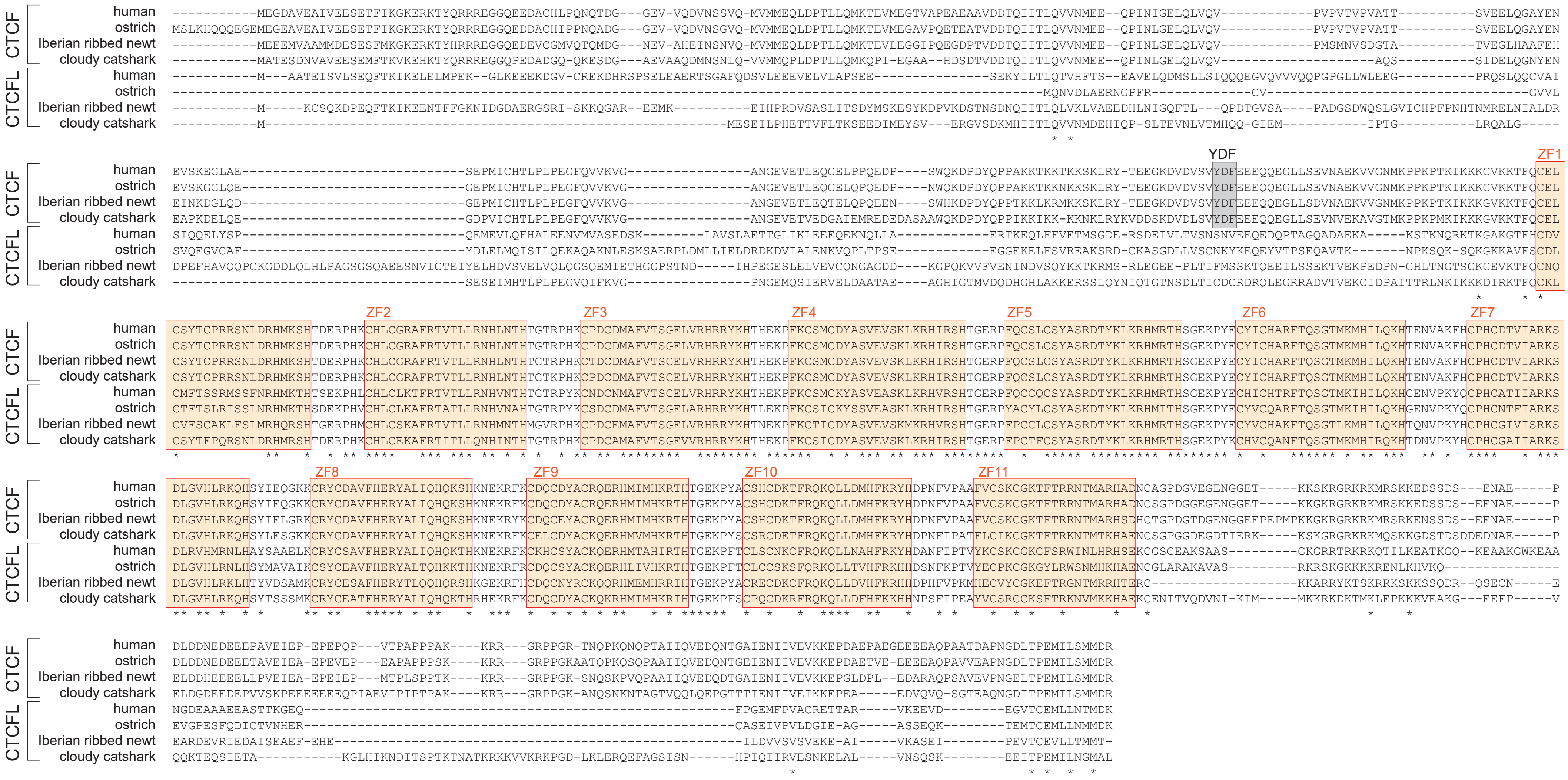

Supplementary Figure S2: Multiple amino acid sequence alignment of vertebrate CTCF and CTCFL. The alignment was generated by MAFFT[1] ver. 7.471 (https://mafft.cbrc.jp/alignment/server/). An asterisk indicates an identical amino acid residue. Zn finger domains (ZFs) were identified by the webserver MOTIF Search (http://www.genome.jp/tools/motif/) and highlighted with colored boxes. The YDF motif that interacts with the SA2-SCC1 subunit of cohesin[2] is highlighted with a gray box. See Supplementary Table 1 for detailed information of the sequences used for the analysis.

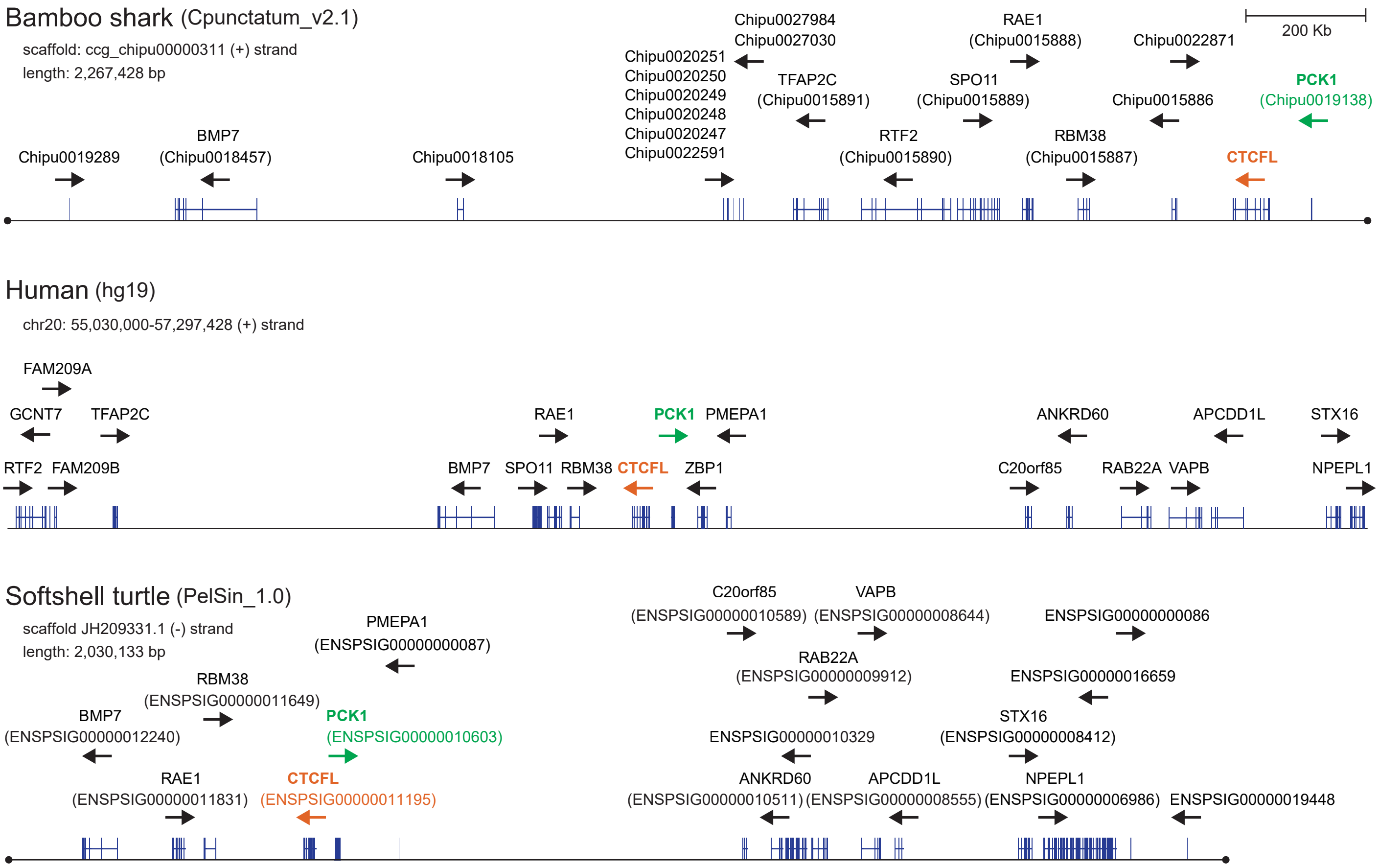

Supplementary Figure S3: Gene synteny conserved among the genomic region containing the *CTCFL* orthologs. The scaffold ccg_chipu00000311 of the bamboo shark genome assembly Cpunctatum_v2.1 and the scaffold JH209331.1 of the softshell turtle assembly PelSin1.0 are shown together with their orthologous gene loci of the human chromosome region 20q13. The black dots indicate scaffold ends.

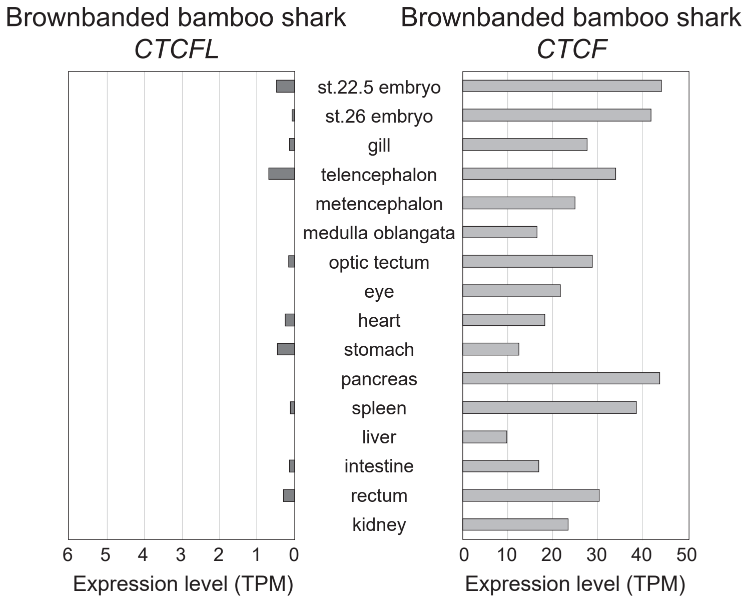

Supplementary Figure S4: Expression profiles of *CTCF* and *CTCFL* genes in bamboo shark tissues. Expression levels of the bamboo shark *CTCF* and *CTCFL* in juvenile tissues and embryos at different developmental stages were quantified in TPM (transcripts per kilobase million mapped reads) values as shown in Figure 5 for the cloudy catshark (see Methods) using reads mapped to the set of coding nucleotide sequences of the bamboo shark (in the file 'Cpunctatum_v1.0.cds.nuc.fna' retrieved from https://figshare.com/projects/sharkgenome1-phyloinfokobe/28863) including that of the CTCFL sequence (see Supplementary Table S1). Bamboo shark embryos were staged according to existing literature[3]. Details of the RNA-seq data used for the analysis are included in Supplementary Table S2.

Supplementary Table 1. Identifiers of the sequences used for phylogenetic analysis.

Group	Species	Gene name	Accession ID	Data source
CTCF	human	*CTCF*	NP_006556.1	NCBI
	softshell turtle	*CTCF*	ENSPSIP00000003796.1	Ensembl
	ostrich	*CTCF*	XP_009685109.1	NCBI
	western clawed frog	*CTCF*	NP_001116268.1	NCBI
	Iberian ribbed newt	*CTCF*	M0105309_PLEWA04	iNewt[†]
	coelacanth	*CTCF*	XP_005992836.1	NCBI
	brownbanded bamboo shark	*CTCF*	Chipu0005442	Squalomix[‡]
	whale shark	*CTCF*	Rhity2000076	Squalomix[‡]
	cloudy catshark	*CTCF*	AYG51206.1	GenBank
	Callorhinchus milii	*CTCF*	XP_007887703.1	NCBI
CTCFL	human	*CTCFL*	NP_542185.2	NCBI
	ostrich	*CTCFL*	see Kadota *et al.*, 2017	Kadota *et al.*[4]
	softshell turtle	*CTCFL*	ENSPSIP00000012493.1	Ensembl
	Iberian ribbed newt	*CTCFL*	M0246129_PLEWA04	iNewt[†]
	brownbanded bamboo shark	*CTCFL*	see Supplementary Data 1	This study
	whale shark	*CTCFL*	see Supplementary Data 1	This study
	cloudy catshark	*CTCFL*	KY883980	GenBank
Cyclostome CTCF	Arctic lamprey	*CTCF*	AQM52683.1	GenBank
	sea lamprey	*CTCF*	ENSPMAP00000004689.1	Ensembl
	inshore hagfish	*CTCF*	see Kadota *et al.*, 2017	Kadota *et al.*[4]
Cyclostome CTCF2	Arctic lamprey	*CTCF2*	AQM52684.1	GenBank
	sea lamprey	*CTCF2*	see Kadota *et al.*, 2017	Kadota *et al.*[4]
Invertebrate CTCF	*Ciona intestinalis*		NP_001104593.1	NCBI
	Oikopleura dioica		CBY12384.1	GenBank
	Branchiostoma belcheri	CTCF-like	XP_019641805.1	NCBI
PCK1	human	*PCK1*	NP_002582.3	NCBI
	opossum	*PCK1*	XP_001377807.2	NCBI
	softshell turtle	*PCK1*	ENSPSIP00000011834.1	Ensembl
	chicken	*PCK1*	NP_990802.1	NCBI
	western clawed frog	*PCK1*	NP_001073036.1	NCBI
	coelacanth	*PCK1*	XP_005990651.1	NCBI
	brownbanded bamboo shark	*PCK1*	see Supplementary Data 1	This study
	whale shark	*PCK1*	see Supplementary Data 1	This study
	cloudy catshark	*PCK1*	see Supplementary Data 1	This study
	Callorhinchus milii	*PCK1*	XP_007882639.1	NCBI

PCK2	human	*PCK2*	NP_004554.3	NCBI
	opossum	*PCK2*	XP_016284237.1	NCBI
	chicken	*PCK2*	NP_990801.1	NCBI
	western clawed frog	*PCK2*	XP_012813768.1	NCBI
	coelacanth	*PCK2*	XP_014352392.1	NCBI
	brownbanded bamboo shark	*PCK2*	PE_TRINITY_DN206856_c3_g2_i6	Squalomix‡
	whale shark	*PCK2*	see Supplementary Data 1	This study
	cloudy catshark	*PCK2*	PE_TRINITY_DN70459_c7_g3_i5	Squalomix‡
Invertebrate PCK1/2	*Ciona savignyi*		ENSCSAVP00000009425.1	Ensembl
	Ciona intestinalis		XP_002128953.1	NCBI
	Branchiostoma belcheri	*PCK*	XP_019640194.1	NCBI
	Branchiostoma floridae		XP_002593974.1	NCBI

†The sequence was obtained from the sequence archive iNewt (http://www.nibb.ac.jp/imori/main/).

‡The sequence was obtained from the elasmobranch sequence archive Squalomix (https://transcriptome.riken.jp/squalomix/).

Supplementary Table S2. RNA-seq libraries used for gene expression analysis.

Species	Tissue	Sex	Library ID	Accession ID
Scyliorhinus torazame	embryo stage 8	unknown	P163_11_1	DRR111753
	embryo stage 17	unknown	P163_20_1	DRR111762
	embryo stage 29	unknown	P180_10_1	DRR111774
	skin	male	P246_77_1	DRR111834
	gill	female	P246_01_1	DRR111777
	telencephalon	female	P246_02_1	DRR111778
	metencephalon	female	P246_03_1	DRR111779
	medulla oblongata	female	P246_05_1	DRR111780
	hypothalamus	female	P246_34_1	DRR111792
	optic tectum	female	P246_32_1	DRR111790
	eye	female	P246_37_1	DRR111795
	atrium of the heart	female	P246_06_1	DRR111781
	stomach	female	P246_07_1	DRR111782
	pancreas	female	P246_08_1	DRR111783
	spleen	female	P246_09_1	DRR111784
	liver	female	P246_13_1	DRR111788
	intestine	female	P246_11_1	DRR111786
	rectum	female	P246_12_1	DRR111787
	kidney	male	P246_40_1	DRR111798
	gallbladder	female	P246_36_1	DRR111794
	testis	male	P246_39_1	DRR111797
	epididymis	male	P246_42_1	DRR111800
	ovary	female	P246_10_1	DRR111785
Chiloscyllium punctatum	embryo stage 22.5	unknown	P192_08_1	DRR111721
	embryo stage 26	unknown	P192_07_1	DRR111727
	gill	male	P246_19_1	DRR111734
	telencephalon	male	P246_20_1	DRR111735
	metencephalon	male	P246_21_1	DRR111736
	medulla oblangata	male	P246_25_1	DRR111740
	optic tectum	male	P246_22_1	DRR111737
	eye	male	P246_26_1	DRR111741
	heart	male	P246_28_1	DRR111743
	stomach	male	P246_14_1	DRR111729
	pancreas	male	P246_29_1	DRR111744
	spleen	male	P246_18_1	DRR111733
	liver	male	P246_17_1	DRR111732
	intestine	male	P246_16_1	DRR111731
	rectum	male	P246_15_1	DRR111730
	kidney	male	P246_30_1	DRR111745

The details of library preparation conditions are found in the Supplementary Table 6 of the publication by Hara *et al*.[5]

Supplementary Data 1. Nucleotide sequences containing the ORFs of the genes analyzed in this study that were manually curated. See Supplementary Table 1 for the other sequences used for the phylogenetic analysis in this study.

>*Chiloscyllium punctatum* CTCFL
TTGGAAACAATGATGGAAAATGAAATTTTACCCCATGATACAACTGTATTTTTGGCAAAGTCAGAAGAGGACATCATGG
AATATTCTGTGGAGCGGGGAGTATCTGACAAAATGCATGTTATCACGTTGCAGGTAGTCAACATGGATGAACACATCCA
GCCCAGCCTGACTGAGGTCAACGTGATGACAATGAACCAACAAGGAGTTGAAATTATTCCAAGTGAGCTGAGGCAGGCC
TTGAGTAGTGAGGGTGAAATCATGCACACTCTTCCACTCCCTGAAGGAGTGCAGGTTTTTAAGGTAGGGCCAAATGGAG
AGATGCAGTATATTGAGAGAATGGAACTGGATGCAGCCCATACTCCAGAAGAATACATAGGCACAGTGATAGAACAATA
CCACGATCCACTTGTCAAGAAAGAGAAAAGGAGTCTGCAGTGCGATGTACAAATTGGGAATAATACAGATGTAACCATT
CATCATTGTAGAGACAGACAACTGGAGGGACTACAGTCGGGTGTAGCTGTTGAAAAAACAACTAGTTCTGCCACTACGG
CTGGACTGAACAAAACTAAAAAGCAAGATATTAAGAAGACATTTCAGTGTAAATTATGTTCACGCACTTTTACACAACG
GTCTAATCTAGACCGTCATATGAGAAGCCATTCAGATGAGCGTCCACACAGATGTCATCTTTGTGAAAAGGCATTCCGA
ACTGTCACCCTCCTGCAAAACCATATCAACACTCATACAGGTACAAGACCACACAAATGTCCTGACTGCGCAATGGCAT
TTGTTACAAGTGGGGAGGTGGTGCGACACAGACGTTACAAGCACACCCATGAGAAACCCTTCAAATGCTCAATCTGCGA
TTATGCTAGTGTTGAGGTAAGTAAACTGAAGCGACACATCCGTTCTCACACCGGGGAGCGTCCTTTCCCGTGCACATTT
TGCAGCTATGCTAGCAGAGATACATACAAGCTGAAGCGACATATGAGAACTCACTCAGGGGAGAAGCCTTACAAGTGCC
ATGTGTGCGAGGCTAATTTTACACAGAGTGGAACGATGAAAATGCACATCCGACAGAAGCACACAGACAATGTGCCCAA
ATACCACTGTCCTCACTGTGATGCCATCATTGCAAGAAAAAGTGACTTGGGTGTCCATTTACGAAAGCAGCATTCCTAC
TCAAGCTTAAGCATGAAGTGTCGTTACTGTGATGCTACATTCCATGAACGCTATGCACTCATCCAACACCAAAAGACCC
ACAGGAATGAGAAACGTTTCAAGTGTGACCAGTGTGATTATGCATGTAAACAGAAACGTCACATGATAATGCATAAACA
TATCCACACTGGGGAAAAGCCATTCAGTTGCTCCCACTGCGATATGCACTTCAGACAGAAGCAGCTCCTGGATTTGCAT
TTCAAGAAGAACCACGATCCAAGTTTCATCCCAGAAGCATACATTTGCTCCCAATGTTGTAAGAGCTTTTCTCGCAAGA
ATGTCATGAAAAGGCATGCTGAGAGATGTGGCAATGTGACGATACAAGATGGAAATCCAAAGTTTATGGCAAAGAAAAG
GAGAAATAGGAGGAATAAATTGGAGTACAACCACAATGCTGAAGCAAAAGGAGATGGACCTTCAGTTCGACTTGAGACT
CAACAACCCATTGAAATAACTGATGAACCTCACGTCAAGAATGCTGCCACATCTCTGACTAAAACAAATGTGATTAAAA
GGGAAAAAATGGATAAAAGAAAATCAGCTGGAATGAAATCAGAGCCACAGGAGTTTACAGGCAGTGTTTCTAACCACAC
AATTGAGGTCATACCAGTTGACAGCAGCAAAGAGCTAACCACAGTGAATAGTCAGCGCAAAAAAACGATCACCCCTGAG
ATGATTCTGAAACGAATGGTCCTGTAA

>*Rhincodon typus* CTCFL
TTGGAAAGAATGACGGAAAGTGAAATTTTTCCCCACGATACAACTGTGTTTTTGGCAAAGTCATACGAAGACATCATGG
AATATGCTGTGGAGCGGGGAGTTTCTGACAAAATGCGCATTATCACATTACAAGTAGTCAACATGGATGAACACATCCA
GCCCAGCCTGACTGAGGTCAACTTGACAATGAACCAACAAGGAACTGAAATGATTCCAAATGGGCTGAGACAGGCGTTG
GGTGGTGAGGGTGAGATCATGCACACTCTTCCACTCCCTGAAGGAGTACAAGTTTTTAAGGTAGGGCCAAATGGAGAGA
TGCAGTGCATTGAGAGAATGGAACTGGATGCAGCCCATACTCCGGAAGAATGCGTAGACACAGTGATAGACCAATACCA
CGAACCTCTTGTCAAGAAAGAGAAAAGTAGTGTGCAGTGTGGTATACAAATTGAGAATAATACAGATGTGACCATTCAT
CATTGTAGAGGCAGACAGCTGGAGGGTCTACAACCAGATGTATCTGTTGAAAAAACTATTAGTCCTGCTAGACTTAACA
AAATTAAGAAGAAAGATGTTAAGAAGACATTTCAGTGTAAACTATGTTCTTATACATTTACACAACGGTCTAATCTAGA
CCGTCACATGAGAAGCCATTCAGATGAGCGGCCACACAAATGTCACCTTTGTGAAAAGGCTTTTCGAACCATCACCCTT
CTGCAAAACCATATAAACACTCATACAGGCACTAGACCACATAAATGCCCTGACTGTGCTATGGCATTTGTTACGAGCG
GTGAGGTGGTGCGACACAGACGTTACAAGCATACCCATGAAAAGCCCTTCAAATGTTCAATCTGCGATTATGCTAGTGT
TGAGGTAAGTAAACTGAAGCGACACATCCGTTCTCACACAGGGGAGCGTCCATTCCCGTGCACATTCTGCAGCTATGCT
AGCAGAGATACATACAAGCTGAAGCGACATATGAGAACTCACTCAGGGGAGAAGCCTTACACATGCCATGTATGCCAGG
CTAATTTCACACAGAGTGGGACAATGAAAATGCACATCCGGCAGAAACACACAGACAATGTGCCCAAATACCACTGTCC
TCACTGTGATGCCATCATCGCAAGAAAAAGTGACTTAGGTGTCCATTTACGAAAGCAGCATTCCTACACAAGCTCGAGC
ATGAAGTGTCGTTACTGTGAAGCTACTTTCCATGAACGCTATGCACTCATCCAACACCAAAAGACGCACAGGAATGAGA
AACGTTTCAAGTGTGACCAGTGTGATTATGCATGTAAACAGAAACGTCACATGATAATGCATAAACGTATCCACACCGG
AGAAAAGCCATTCAGCTGCTCCCAGTGTGATAAGCAGTTCAGACAGAAGCAGCTTCTGGACTTGCATTTCAAGAAGCAT
CACGATCCAAGTTTCATCCCAGAGGCATACATTTGCTCCCAGTGTTGTAAGAGCTTTACTCGCAAGAATGTCATGAAAA
GGCATGCTGAGAGATGTGGCAATGTGACAATACAAGATGGAAGTGCAATGTTTATGCCAAAGAAAAGGAGGATCAAGAA
GAATAAATTGGAGTACAAACACAATGCTGAAGCAAAAGGAGATGAACCTCCAGTTGGACTTGAGACTCAACAATCCATT

GAAGTAACTGATGACTGTCACATCAAGAATGATGTCACATCACCGACTAAAACAAATGTGATTAAAAGGAAAAAAATGG
TTAAAGGAAAATCAGCTGGAGTGAAGTTGGAGCCACAGGAGTTTGCAGGCAGTGTTTCTAACCACACAGTTGAGATCAT
ACCAGTTGACAGCAACAAAGAGCTAACCACAGTGAATGGTCAGTGCAAAAAAACAATCACCCCTGAGATGATCCTGAAA
CGAATGGTCTTGTAA

>*Chiloscyllium punctatum* PCK1
ATGGCCCCTCAACTCCAATCTCAGCCCCAGCACCCTCTGAATGTGGTTCAGGGTGATCTGAGTGCCTTGAGCCCTGCAG
TGAGAGACTTTGTTGAACAAAGTGCAAAGCTATGTCAACCAGACAAGATCCACATCTGTGATGGGAGTGAAGAAGAGAA
CAGGCAGCTTCTGTGCCTGATGGAAGAGCAAGGGATAATCAAACGCTTACCTAAGTGTCAGAACTGCTGGTTGGCTCGC
ACAGACCCCAGAGATGTTGCTCGTGTGGAGAGTAAAACTGTAATTGTGACTCCTGAACAGAGAGATACCATTCCCATAG
CAAAGAGCGGGGTCAGCCAACTTGGGCGCTGGCTGTCGGAGAAGGAATTTGAAAAGGCACTCGCTCTACGTTTCCCAGA
ATGTATGAAAGGCCGTACAATGTACCTGATTCCATTCAGCATGGGACCTGTGGGATCACCTTTGTCCAAGATCGGGATC
CAGTTGACAGATTCTCCTTATGTCGTGGCAAGTATGAGGATCATGACTCGCATGGGATCAGCTGTACTGGAGACCCTGG
GCAATGGCCAGTTTGTCAAATGCCTTCACTCAGTCGGATGCCCTTTACCTCTAAAGAAACCACTGGTCAATAACTGGGC
CTGCAATCCTGAGCTAACACTGGTTGCCCACATCCCTCAGCGCAGGGAGATTATTTCATTCGGGAGTGGTTATGGAGGT
AACTCGCTGCTGGGAAAGAAGTGTTTTGCTCTCCGCATTGCCTCCCGGATTGGTGAAGAAGAGGGCTGGCTTGCTGAGC
ACATGCTGATCATGGGAGTAACCAATCCTAAAGGCCAGAAAAAGTACCTTGCTGCTGCATTCCCTAGCGCTTGTGGGAA
AACAAACTTGGCCATGATGAATCCAACATTACCCGGTTGGAAAGTCGAGTGTGTGGGAGATGACATCGCCTGGCTGAAA
TTCGATGATCAAGGTAAACTAAGAGCAATCAACCCAGAAAATGGCTTCTTTGGTGTAGCTCCTGGGACATCTATGAAGA
CAAATCCAAATGCAATGCACACCATCAGCAAGAACACCATCTACACCAATGTGGGAGAAACCAGTGATGGGGGTGTTTA
CTGGGAAGGAATCGATGAAACTCTGCCCCCAGGTATCACAGTGACTTCATGGAAAAACAAAAAATGGAGTCCTGATGAT
GGAGAACCATGTGCACACCCCAACTCCAGGTTCTGTACTCCTGCCAGACAGTGTCCAATTATTGACCCAAACTGGGAGT
CTCCTGAAGGGGTCCCCATTGAAGGAATTATCTTCGGAGGACGGAGACCAAAAGGTGTCCCTCTGGTTTATGAAGCTTT
TAACTGGCAGCATGGAGTTTACATTGGGGCATCCATGAGATCAGAAGCTACAGCTGCAGCAGAACACAAAGGTAAGATC
ATTATGCATGACCCTTTCGCCATGCGGCCTTTCTTTGGCTACAACTTTGGGAGGTACCTGTCGCACTGGCTCAGCATGG
AGCACAGGACCTGCTCAAAACTTCCCAAGATCTTCCACGTCAACTGGTTCCGCAAAGATAAACAGAACAACTTCCTGTG
GCCAGGTTTCGGGGAGAACTGTCGAGTTCTGGAGTGGATGTTCCGGAGAATCGAGGGAGAGGACTGCGCCAAGATGTCT
CCTTTGGGCTACATTCCCGCAGACGGTGCCCTCAACCTCAATGGTCTCAGCCACGTGAACATGGACGAAATCTTCAGTA
TTGACAAAGAATTCTGGGAAGAGGAAATCAAAGAGATCCGAAAATACTTTGACGATCAGGTCAACACTGACCTTCCCTG
TGACGTGGCCAATCAGCTGTTGCAGTTGGAACAAAGAATAAATCGGTTGTAA

>*Rhincodon typus* PCK1
ATGGCCCCTCAACTCCAATCTCAGCCCCAACACCCTCTGAATGTGGTTCAGGGAGATCTGAATGCCTTGAGCCCTGCAG
TGAGAGACTTTGTTGAACAAAGTGCGAAGTTATGTCAACCAGACAAGGTCCACATCTGTGATGGGAGTGAAGATGAGAA
CAGGCAGCTTCTGTGCCTGATGGAAGAGCAAGGGATAATCAAACGCTTAACCAAGTATCAGAACTGCTGGTTGGCTCGC
ACAGACCCCAGAGACGTTGCTCGTGTGGAGAGTAAAACCGTAATTGTGACCCCTGAACAGAGAGATACCATTCCCATAC
CAAAGAGTGGGGTCAGCCAGCTCGGGCGCTGGCTGTCAGAGAAGGAGTTTGAAAAGGCATTCACTTTACGTTTTCCAGA
ATGTATGAAAGGCCGTACAATGTACCTGATTCCATTCAGCATGGGACCTGTGGGTTCACCTTTGTCCAAGATCGGGATC
CAGTTGACAGATTCACCTTACGTTGTGGCAAGCATGAGGATCATGACTCGCATGGGATCGGCTGTACTGGAGACCCTGG
GCAATGGCCAGTTTGTCAAATGCCTTCATTCAGTTGGATGCCCTTTACCACTAAAGAAACCACTGGTCAATAACTGGGC
CTGCAATCCTGAGCTGACACTGGTCGCCCACATCCCTCAGCGCAGGGAGATTATTTCATTCGGGAGTGGTTATGGAGGT
AACTCACTGCTGGGAAAGAAGTGCTTTGCTCTGCGCATTGCCTCCAGGATTGCTGAAGAAGAGGGCTGGCTTGCTGAAC
ACATGCTGATCATGGGAGTAACCAACCCCAAAGGCCAGAAGAAGTACATTGCTGCTGCGTTCCCTAGTGCTTGCGGAAA
AACAAACTTGGCCATGATGAATCCGACATTACCCGGTTGGAAAGTCGAGTGTGTGGGAGATGATATCGCCTGGCTGAAG
TTCGATGAGCAAGGTAAACTGAGAGCAATCAACCCAGAAAATGGCTTCTTTGGTGTAGCTCCTGGGACATCTAACAAGA
CAAATCCAAATGCAATGCAAACCATCAGCAAGAACACCATCTTCACCAACGTGGCAGAAACCAGCGATGGGGGTGTTTA
CTGGGAGGGAATTGATGAAACTCTGCCCCCCGGCATCACAGTGACTTCATGGAAAAACAAAATATGGAGTCCTGGCGAT
GGAGAACCATGTGCACACCCCAACTCCAGGTTCTGTACTCCTGCCAGACAGTGTCCAATCATTGACCCAAATTGGGAGT
CTCCTGAAGGGGTTCCCATTGAAGCAATTATCTTCGGAGGACGGAGACCAAAAGGCGTCCCTCTGGTTTATGAAGCTTT
TAACTGGCAGCATGGAGTTTATGTTGGAGCATCCATGAGATCAGAGGCTACAGCTGCAGCAGAGCACAAAGGTAAGGTC
ATTATGCATGACCCTTTCGCCATGCGGCCTTTCTTTGGCTACAACTTTGGGAAGTACCTGTCACACTGGCTCAGTATGG
AGCACAGGACATGCTCGAAACTCCCCAAGATCTTCCACGTCAACTGGTTCCGCAAAGACAAGCAGAACAACTTCCTGTG
GCCGGGTTTCGGGGAGAACTGTCGAGTCCTGGAATGGATGTTCCGGAGAATCGAGGGAGAGGACTGTGCCAAACTGTCT
CCTTTGGGCTACATTCCTGCAGATGGTGCCCTGAATCTCAACGGTCTCAGCTACGTGAACATGGACGAAATCTTCAGTG
TTGAGAAGGAATTCTGGGAAGAGGAAATCAAAGAGATCCGAAAATATTTTGACGACCAGGTCAACATTGACCTTCCCTG

TGATGTGGCCAATCAGCTGCTACAGTTGGAACAAAGAATAAATCGGTTGTAA

>*Scyliorhinus torazame* PCK1
ATGGCACCACAGCTCCAGCCTCCGCCCCGGCTCCCTCTGAATGTGGTTCAGGGGGATCTGAACACGCTGAGCCCTGCAG
TGAGAAACTTTGTTGAAGAGAATGCACAGCTGTGTCAGCCGAACAACATCCATATCTGTGATGGCAGTGAGGAAGAAAA
CCGGCAGATTTTGTCCCTGATGGAAGAGCAAGGCATGATCAAACGTTTGCGCAAGTATCAGAACTGCTGGTTGGCTCGT
ACAGACCCCAGGGATGTTGCTCGTGTGGAGAGTAAAACTGTGATTGTGACCCCTGAACAGAGAGACACCATTCCTGTAG
CAAAGAGTGGTGTCAGCCAGCTCGGGCGCTGGCTGTCGGAGAAGGAATTTGAAAAGGCATTCAATGCCCGATTCCCAGA
GTGTATGAAAGGCCGTACAATGTACCTGATTCCATTCAGCATGGGGCCTGTGGGATCACCTCTGTCCAAGATCGGGATT
CAGCTGACTGATTCACCTTACGTCGTGGCTAGCATGAAGATCATGACTCGCATGGGATCAGCTGTACTGGAGACCCTGG
GGAATGACCAGTTCGTCAAATGCCTTCATTCAGTCGGGTGCCCTTTGCCGCTAAAGAGGCCACTGGTCAATAACTGGGC
CTGCAATCCCGAGCTGACTCTGGTCGCCCACATACCTCAGCGCAGGGAGATTGCTTCATTTGGAAGCGGATATGGAGGT
AACTCACTGCTGGGGAAGAAGTGCTTCGCTCTCCGCATCGCATCCAGGATTGCTAAAGAAGAGGGCTGGCTGGCTGAGC
ACATGCTGATCCTGGGAGTGACCAATCCCAAAGGGCAGAAGAAGTACATTGCTGCTGCCTTCCCCAGTGCTTGTGGGAA
AACAAACATGGCCATGATGAATCCGACATTACCCGGCTGGAAGGTGGAGTGTGTGGGAGATGACATCGCCTGGATGAAA
TTTGATGAACAAGGTAACCTGAGGGCAATCAACCCAGAAAATGGCTTCTTTGGTGTAGCTCCTGGGACGTCTGTAAATA
CAAACCCAAACGCAATGAAAACCATCAGCAAGAACACCATTTTCACCAATGTAGCAGAGACCAGCGATGGGGGTGTTTA
CTGGGAAGGAATCGATGAAAATCTGCCCCCCGGTGTCACATTAACTTCCTGGAAAAACAAAGAGTGGAGACCAGAGGAT
GGAGAACCATGTTCACACCCCAACTCCAGGTTCTGTACTCCTGCCGGACAATGTCCGATCATTGACCCAGACTGGGAGT
CTCCTGAAGGAGTTCCCATTGAAGGAATTGTCTTCGGAGGACGGAGGCCAAAAGGAGTCCCTCTGGTGTATGAAGCTTT
CAACTGGCAGCATGGGGTTTTCATCGGAGCAGCCATGAGATCAGAGGCTACAGCTGCAGCAGAACACAAAGGGAAGGTC
ATTATGCACGACCCTTTCGCCATGCGGCCTTTCTTTGGATACAACTTTGGCAGATACCTATCACACTGGCTCAGCATGG
AGCACAGGGCCGGCTCAAAACTTCCCAAGATCTTCCACGTCAACTGGTTCCGCAAAGATAATCAGAACAACTACTTGTG
GCCGGGCTTTGGGGAGAACTGTCGAGTCCTGGAGTGGATGTTCCGGAGAATCGAGGGGGAGGACTGCGCCAAGCTGTCT
CCTTTGGGCTTCATTCCTGCAGACAACGCCCTTAACCTCAACGGCCTCGGCGTCGTGAACATGGACGAACTCTTCAGTG
TTGAGAAAAAATTCTGGGAAGAGGAAATAGAAGCCATCAGAAAATATTTTGAAGATCAGGTCAACATCGACCTTCCCAG
TGACGTGGCCAATCAGCTGCTGCAGTTAGGACAAAGAATAAGTCGGGTGTAA

>*Rhincodon typus* PCK2
ATGTGTTCGATCTACGCTCGGATATCCCGATTCTCCACGTTCTCACTCAGATGTCTGACTGAGCGGTGGCGATGCCACC
TGCCCACCCTGCCCAGACGCTGCGCCCACTCTCCGCCCCTGGCGAGCAGGGATGTGTCGGGTCTGCCGGCCGACGTGCG
GGAGTTTATAGAGTCCAGCGCCCGCCTGTGCCGGCCCAGCCACGTCCACGTCTGCACGGGCACTGAGGAGGAGAACTCG
GCCATTCTCGCGCAGCTTTCGAGGGATGGGCTCATTAAGCAGCTGCCAAAATATGAGAACTGCTGGCTGGCATGGACAG
ACCCGAGAGACGTGGCCCGTGTGGAGAGTAAGACGGTGATAGTAACGGAGAACCGAAGGGACACCATACCCACTCCCAG
CGACGGGAACCCCAGCCAATTGGGAAACTGGATGGCCATGGAGGAATTCGACAGGGCAGTGGCACAGAGGTTCCCAGGG
TGTATGGCAGGTCGGACGATGTACGTGATCCCCTTCAGCATGGGTCCCATCGGATCCCCTCTCTCCAAGATCGGAATCC
AGCTGACAGACTCTGCCTACGTAGTGGCCAGCATGCACATCATGACCCGCATGGGCACCCCGGTCCTGGCAGCGCTGGG
CACAGGAGAGTTTGTCAAGTGTTTGCACTCGGTGGGATACCCGCTGAGCTCGGAGGGTGGCGGGGGCCCCGCCTGGCCA
TGTAATCCCGAGAAAACCCTGATCATGCACGTCCCAGACCGTCGGCAGATCCTGTCGTTCGGCAGCGGCTATGGCGGGA
ACTCCCTGCTCGGGAAGAAGTGTTTCGCTCTCAGGATCGCCTCGCGCATCGCCAAGGACGAAGGCTGGCTGGCCGAGCA
CATGTTGATCCTGGGAGTCACCAATCCCACGGGCCAGAAGAAATACATCGCTGCAGCGTTCCCAAGTGCCTGTGGGAAA
ACCAACATGGCCATGATGCAGCCCTCCCTCCCCGGGTGGAAAGTCGAGTGCGTGGGGGACGACATTGCCTGGATGAGGT
TCGACAGTCAGGGGCGTCTGCGAGCGATCAACCCGGAAAATGGCTTCTTTGGGGTGGCGCCAGGAACATCCATGCAGAC
CAACCCCAACGCCATGGAGACCATCTCCAGGAACACGGTCTTCACCAACGTGGGGGCCACCAGCGACGGGGGCGTTTAC
TGGGAGGGAATTGACCAGCCCCTCCCTCCCGGCGTCACCATCACCTCCTGGACCGGGAAGCCCTGGAGACCAGGTGACA
AGGAGCCCTGTGCCCACCCCAACTCGCGGTTCTGCGCTCCCGCCAGGCAGTGCCCCATCATGGACGAGAACTGGGAGTC
GCCCGAGGGGGTCCCAATCGATGCCATCGTATTCGGGGGCAGGAGGCCCGAAGGCGTGCCCCTCGTGTACGAGGCCTAC
AGCTGGCGACACGGAGTGTTCGTTGGGGCCGCCATGAGATCAGAGTCAACAGCAGCAGCTGAGCACAAAGGGAAGACCA
TCATGCACGACCCCTTCGCCATGAGGCCGTTCCTGGGCTATAACTTTGGAGATTACCTCCAGCACTGGCTGTCGATGGG
GGAGAAGGAGGGGGCCAGCTTGCCTCGCATCTTCCACGTCAACTGGTTCCGGAAGGGTCGGGATGGCACCTACCTGTGG
CCGGGCTTCGGGGAGAACTGCCGGGTGCTCGACTGGATCTGCCGGAGGGTGGATGGCCAGGCCGGAGCGGAGGGTACAG
CGGTGGGTTACGTGCCATCTCCGGGCTCGCTGGACCTGAGCGGGCTCCGGGGGGTGAGGGCGGCCGAGCTGTTCGAGCT
GCCCCGGGCCTTCTGGGAGAGGGAGGCCTCCGACCTGCGCTCCTACCTCACCATCCAGGTCAACCGCGACCTGCCACAG
GCCATCAGCGACGAGCTGGAGGGGTTGGAAAGGCGGCTGGGGGCTATGTAA

References for Supplementary Information

1. Katoh, K. & Standley, D.M. MAFFT multiple sequence alignment software version 7: improvements in performance and usability. *Mol Biol Evol* **30**, 772-80 (2013).
2. Li, Y. *et al.* The structural basis for cohesin–CTCF-anchored loops. *Nature* **578**, 472-476 (2020).
3. Onimaru, K., Motone, F., Kiyatake, I., Nishida, K. & Kuraku, S. A staging table for the embryonic development of the brownbanded bamboo shark (*Chiloscyllium punctatum*). *Dev Dyn* **247**, 712-723 (2018).
4. Kadota, M. *et al.* CTCF binding landscape in jawless fish with reference to Hox cluster evolution. *Sci Rep* **7**, 4957 (2017).
5. Hara, Y. *et al.* Shark genomes provide insights into elasmobranch evolution and the origin of vertebrates. *Nat Ecol Evol* **2**, 1761-1771 (2018).
